# Seasonal variability shapes resilience of small-scale fisheries in Baja California Sur, Mexico

**DOI:** 10.1371/journal.pone.0182200

**Published:** 2017-08-04

**Authors:** Kara E. Pellowe, Heather M. Leslie

**Affiliations:** 1 Darling Marine Center, University of Maine, Walpole, Maine, United States of America; 2 Ecology and Environmental Sciences Program, University of Maine, Orono, Maine, United States of America; 3 School of Marine Sciences, University of Maine, Orono, Maine, United States of America; International Nutrition Inc, UNITED STATES

## Abstract

Small-scale fisheries are an important source of food and livelihoods to coastal communities around the world. Understanding the seasonality of fisheries catch and composition is crucial to fisheries management, particularly in the context of changing environmental and socioeconomic conditions. While seasonal variability directly impacts the lives of fishers, most fisheries studies focus on longer-term change. Here we examine seasonal variability in the small-scale fisheries of Baja California Sur, Mexico based on 13 years of government fisheries data. We investigate how four fisheries indicators with direct relevance to ecological resilience–magnitude and variance of landed fish biomass, taxon richness and the proportion of top-trophic-level taxa in total catch–vary within and among years and at multiple spatial scales. We find that these resilience indicators vary both seasonally and spatially. These results highlight the value of finer-scale monitoring and management, particularly for data-poor fisheries.

## Introduction

Small-scale fisheries provide food security, livelihoods, and other valuable services to coastal communities around the world [1]. However, the resilience of these coupled social-ecological systems is threatened by rising fishing pressure, environmental variability and other forms of disturbance [1]. Resilience is the ability of a system to maintain functioning in the face of disturbance [2], and changes in the resilience of small-scale fisheries can impact the delivery of valuable services, including both food provision and employment [3].

Ecological and social resilience are linked where human communities depend on natural systems, and resilience of social-ecological systems (SES), including fisheries, is enhanced where ecosystem complexity permits diverse resource dependency and adaptive capacity in coastal communities [4]. As social-ecological resilience depends both on resilience of ecosystems and of the institutions governing resource use, investigating ecological resilience is a key step in operationalizing SES resilience [4].

Both theory and empirical studies provide significant evidence of how ecosystem properties contribute to resilience of ecosystems in response to perturbation. For example, diverse assemblages of fished species increase response diversity (*i*.*e*., the diversity of responses to environmental change) and functional redundancy (*i*.*e*., the ability of more than one taxon to perform the same ecological role), whereas high variability in biomass and changes in trophic composition of fished taxa can portend a loss of resilience [5–7]. A loss of biodiversity and a reduction in populations of top-trophic-level taxa may reduce ecological resilience and in turn, resilience of the human communities that rely on natural systems. Earlier authors have suggested a number of ecological resilience indicators, including taxon diversity [6,8], mean trophic level [7], variance in biomass [9], and spatial complexity [10].

While previous empirical fisheries studies have examined changes in ecological variables with direct relevance to resilience over longer time scales [11,12], few have focused on seasonal variation. Long-term changes in resilience indicators can provide a proxy for ecosystem health and the well-being of associated human communities [13], while seasonal changes in these indicators may indicate how human communities adapt to environmental change, and how SESs persist on shorter time scales [14]. Seasonal variability is often easier for fishers to observe than longer-term change, and is directly relevant to the everyday experiences and adaptation strategies of individuals and coastal communities [15].

In this study, we investigate seasonal changes in several potential indicators of resilience in a model fisheries SES in Baja California Sur (BCS), Mexico. BCS borders the Gulf of California, a region renowned for its rich biodiversity and highly productive fisheries. Fishing activities in the Gulf region account for nearly 70% of Mexico’s total annual catch [16]. However, despite increasing catches since 1950, catch-per-unit-effort (CPUE) has decreased since 1980 [17]. In addition to declining CPUE, previous studies on BCS’ small-scale fisheries have documented a decrease in the biomass of top predators over time [17,18], a phenomenon known as “fishing down” the food web [19]. An ecosystem that has been “fished down” is often characterized by a reduction in the mean trophic level of taxa and a loss of top predators and diversity from the system [12,19]. Although catch of upper-trophic-level taxa can remain high even where there is overfishing, declines in the proportion of these taxa in total catch can signal unsustainable harvest and loss of ecological resilience [7].

Reduced diversity of fished taxa may also impact the socioeconomic resilience of fishing communities in BCS to seasonal environmental variation, as many fishers in this region rely on different taxa in different seasons, and in some instances, travel seasonally to different locations within BCS to fish [15,20]. Despite evidence that seasonal dynamics affect fishing activities in this region, seasonality of landings has not been quantified. In addition to seasonal variability, the coast of BCS also includes substantial variety in oceanographic conditions and marine habitats. The Pacific and Gulf of California coasts host distinct oceanographic regions [21], and marine habitats and coastal SESs vary at the within-BCS scale [20].

In this study, we use historical landings data to examine temporal and spatial trends in ecological indicators related to the total biomass and composition of catches reported by small-scale fisheries in BCS, Mexico. Fisheries-dependent data necessarily integrate both ecological and social information, and we use them to investigate how four variables known to be linked with ecological resilience in this and other fisheries-associated SESs (*i*.*e*., magnitude and variance of landed biomass; taxon richness of reported catches; and the proportion of top-trophic-level taxa in reported catches) vary seasonally at two spatial scales: the state of BCS, Mexico and local fishing offices (LFOs) within BCS. We investigate variation in these indicators as a necessary first step to understand ecological resilience of the social-ecological systems associated with marine fisheries in BCS, Mexico. The results of this analysis will enable us to then develop a more informed investigation of the social and ecological characteristics of these SESs that contribute to resilience. The results also will contribute information necessary for crafting appropriately scaled management strategies, in order to sustain these fisheries, and the human communities that depend on them. We hypothesize that:

1. The four variables–magnitude and variance of landed biomass; taxon richness of reported catches; and the proportion of top-trophic-level taxa in reported catches–exhibit spatial variation within the state of BCS.
2. These potential indicators of ecological resilience also vary seasonally (within years) at the scales of BCS and individual LFOs.
3. These potential indicators are correlated in ways consistent with theory, *e*.*g*., those LFOs characterized by high taxon richness also will exhibit low variance of landed biomass and high proportions of top-trophic-level taxa [6,7].

## Materials and methods

### Data source

We use landings data from the Mexican National Aquaculture and Fishing Commission [22] to assess ecological resilience of BCS’ small-scale fisheries. These landings are from 588 small-scale (*i*.*e*., artisanal) fisheries and 10 LFOs within BCS. LFOs are local branches of the Mexican National Aquaculture and Fishing Commission, CONAPESCA, where fishers report their catch on a daily to weekly basis [20]. Each report contains information on the dates of capture, the office where the report was submitted, the fishery type (artisanal; industrial; or aquaculture), names of taxa captured and total biomass and price paid for each taxon. We selected only those fisheries within the “artisanal” category. All fisheries in the dataset are commercial, and fisheries management occurs at the scale of the state. Small-scale fishers typically operate from *pangas*, small (6–8m) fiberglass boats powered with outboard motors, working together in groups of two to three people. Fishing gear varies depending on taxa targeted, but includes gillnets, hook and line, traps, and hookah rigs for diving [20].

### Data preparation

These data were transcribed, cleaned and aggregated into a database by the Scripps Institution of Oceanography’s Gulf of California Marine Program at the University of California San Diego [23]. Cleaning included standardizing common Spanish and scientific taxon names from the descriptive names given on individual landings reports, and assigning each taxon a trophic level, based on life history characteristics. Trophic levels were assigned according to the following: 1 = primary producer, 2 = herbivore, 3 = carnivore, 4 = piscivore. The coarseness of these trophic levels (in contrast to Fishbase, for example) is driven by the data, as many taxa are not identified to species.

The common Spanish names provided the most refined level of taxon identification in this dataset. Where multiple Spanish common names exist for the same taxon, data for all common names were combined so that one complete record existed per taxon. Spanish common, scientific and English common names of top taxa can be found in Table 1.

**Table 1:** Names of top taxa by biomass and value.

Common Spanish name	Scientific name	English common name
Abulon amarillo	*Haliotis corrugata*	Corrugated abalone
Abulon azul	*Haliotis fulgens*	Green abalone
Alga gelidium	*Gelidium robustum*	red seaweed
Almeja catarina	*Argopecten circularis*	Catarina scallop
Almeja chocolata	*Megapitaria squalida*	Chocolate clam
Almeja concha espina	*Spondylus spp*	spiny clam
Almeja navaja	*Tagelus californianus*	California jackknife clam
Almeja pata de mula	*Anadara spp*	blood clam
Almeja pismo	*Tivela stultorum*	Pismo clam
Almeja rosa	*Chione undatella*	Pink clam
Angelito	*Squatina californica*	Pacific angelshark
Barrilete / Barrilete rayado	*Katsuwonus pelamis*	Skipjack tuna
Barrilete negro	*Euthynnus lineatus*	Black skipjack tuna
Bonito	*Caranx caballus*	Green jack
Botete	*Sphoeroides spp*	pufferfish
Cabaicucho	*Diplectrum pacificum*	Inshore sand perch
Cabrilla pinta	*Mycteroperca prionura*	Sawtail grouper
Calamar / Calamar cabeza / Calamar gigante	*Dosidicus gigas*	Giant squid
Callo de hacha	*Atrina spp*	scallop
Camaron	*Litopenaeus spp*	shrimp
Camaron blanco	*Litopenaeus vannamei*	Whiteleg shrimp
Camaron café	*Farfantepenaeus californiensis*	Yellowleg shrimp
Camaron japones	*Sicyonia dorsalis*	Lesser rock shrimp
Cangrejo	*Cancer spp*	crab
Cangrejo moro	*Calappa spp*	box crab
Caracol chino	*Hexaplex spp*	sea snail
Caracol panocha	*Astraea undosa*	Wavy turban snail
Cardenal	*Paranthias colonus*	Pacific creolefish
Charrito	*Trachurus symmetricus*	pacific jack mackerel
Chile / Caiman	*Synodus spp*	lizardfish
Cochito	*Balistes polylepis*	Finescale triggerfish
Cornuda	*Sphyrna spp*	hammerhead shark
Corvina	*Cynoscion spp*	seatrout
Dorado	*Coryphaena hippurus*	Mahi mahi
Erizo rojo	*Strongylocentrotus franciscanus*	Red sea urchin
Erizo morado	*Strongylocentrotus purpuratus*	Purple sea urchin
Estacuda	*Hyporthodus niphobles*	Star-studded grouper
Extranjero	*Paralabrax auroguttatus*	Goldspotted sand bass
Garropa	*Mycteroperca xenarcha*	Broomtail grouper
Guachinango	*Lutjanus peru*	Red snapper
Jurel	*Seriola lalandi*	Yellowtail
Mantarraya	*Dasyatis spp*	stingray
Langosta	*Panulirus spp*	lobster
Langosta azul / Langosta caribe	*Panulirus inflatus*	Blue spiny lobster
Langosta de agua dulce	*Cherax quadicarinatus*	Redclaw crayfish
Langosta roja	*Panulirus interruptus*	California spiny lobster
Lenguado	*Paralichthys spp*	flounder
Lunarejo	*Lutjanus guttatus*	Spotted rose snapper
Mantarraya / Mantarraya aleta	*Dasyatis spp*	stingray
Marlin	*Makaira spp*	marlin
Mero	*Epinephelus spp*	grouper
Ojoton	*Caranx sexfasciatus*	Bigeye
Ostion	*Crassostrea spp*	oyster
Ostion de roca	*Crassostrea iridescens*	Rock oyster
Ostion japones	*Crassostrea gigas*	Pacific oyster
Pampano	*Gnathonodon spp*	trevally
Pargo / Pargo amarillo / Pargo alazan	*Lutjanus argentiventris*	Yellow snapper
Pepino de mar	*Isostichopus fuscus*	Sea cucumber
Perico	*Scarus spp*	parrotfish
Pez espada	*Xiphias gladius*	Swordfish
Pierna	*Caulolatilus princeps*	Ocean whitefish
Pulpo	*Octopus spp*	octopus
Sierra	*Scomberomorus sierra*	Mackerel
Tiburon azul	*Prionace glauca*	Blue shark
Tiburon coludo	*Alopias spp*	thresher shark
Tiburon sedoso	*Carcharhinus falciformis*	Silky shark
Tiburon volador	*Carcharhinus limbatus*	Blacktip shark
Tintorera	*Galeocerdo cuvier*	Tiger shark
Tripa	*Mustelus spp*	smooth hound shark
Verdillo	*Paralabrax nebulifer*	Barred sand bass
Zorro	*Alopias vulpinus*	Common thresher shark

These taxa are among the most important–by both biomass and value–landed by small-scale fishers in BCS from 2001–2013, based on data collected by CONAPESCA. This list includes top taxa by biomass and value for each office, as well as taxa important at the level of BCS. It is not comprehensive of all taxa in the dataset. Please see Methods for details. Where multiple Spanish names exist for the same taxon, they are listed under “Common Spanish name” and separated by a forward slash. English common names are capitalized where they are species-specific; nonspecific English common names are lowercase.

**Fig 1:**
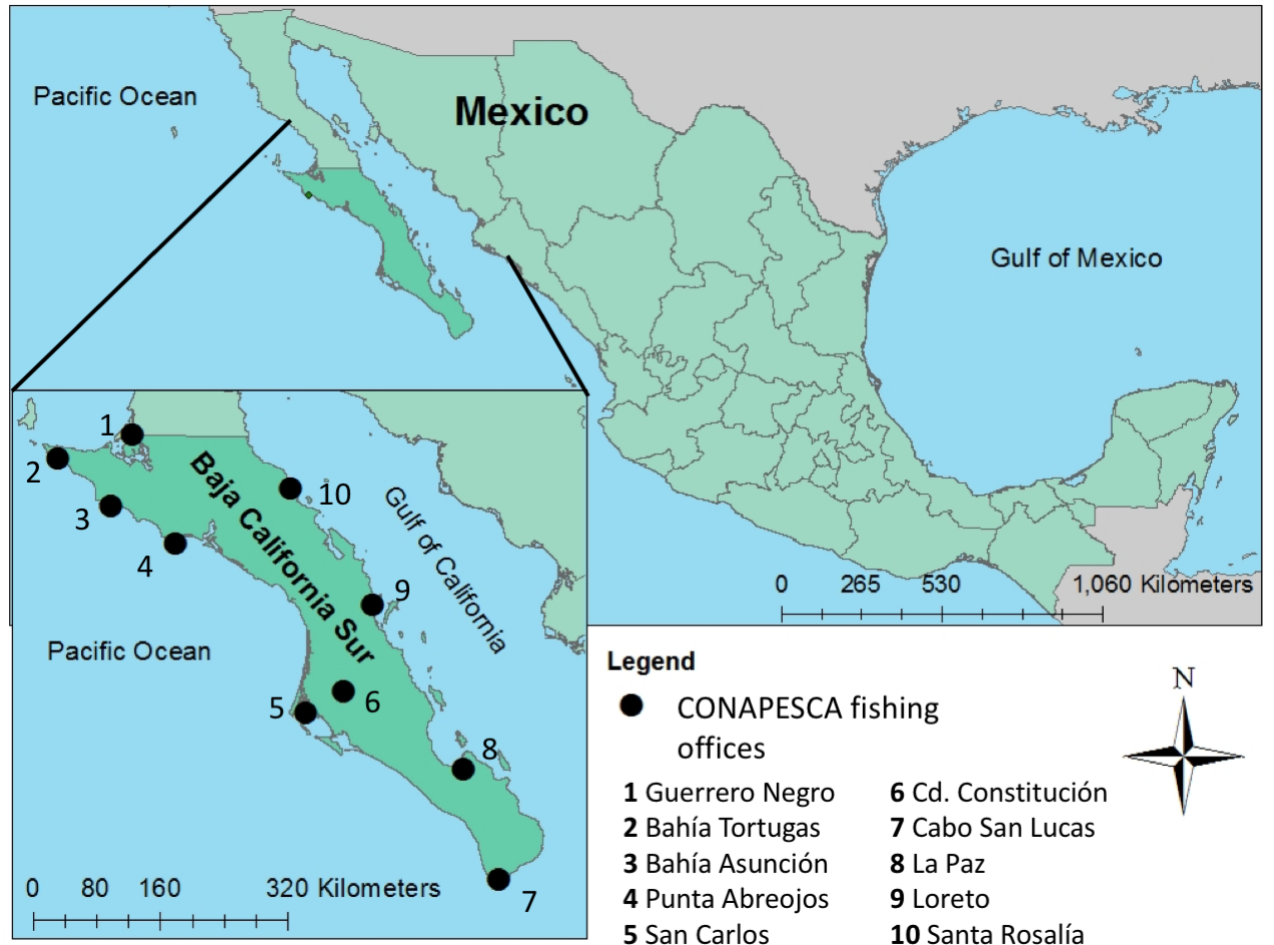
Map of Baja California Sur. Map showing the 10 fishing offices in this study. Reprinted from Pellowe unpub., under a CC BY license, with permission from Kara Pellowe, 2016.

We accessed the data using the visualization and data analytics software Tableau, and prepared data for analyses in both Tableau and Excel (Tableau 9.0, Tableau Software Inc.; Excel 14.4.2, Microsoft Corporation 2010). Data were filtered by year, state, LFO and fishery type. We restricted our analyses to BCS small-scale fisheries landings from 2001 to 2013, because this was the time period where reporting was consistent, with all LFOs reporting catch for each month. Ten LFOs were used in analyses (Fig 1). Landing reports with missing information on month, year or name of taxa (where genus could not be determined) were excluded. While some data were reported daily, these reports were not consistent enough to enable finer scale temporal analyses; therefore we restricted our analyses to the monthly scale. Data were disaggregated by month and year for all resilience indicators except variance in landed biomass, for which only annual data were used.

Resilience variables were calculated as follows: total biomass as the sum of reported biomass; variance in biomass as the square of the standard deviation of the values of biomass for each month in a given year; taxon richness as the number of distinct species reported; and proportion of top-trophic-level taxa as the total predator biomass (summed biomass of taxa with assigned trophic levels 3 and 4) divided by the total biomass of landings. These were calculated for each LFO and month in the dataset, except for variance in biomass, which was calculated for each LFO and year. Data for total biomass and variance in biomass were normalized with a natural log transformation to reduce skewedness.

### Analyses

#### Spatial and temporal trends in resilience indicators

To test the effect of intra-annual trends at the state (Baja California Sur) and LFO spatial scales, we performed an ANOVA with the independent variables LFO and month, and the interaction term LFO*month for each resilience indicator except variance in biomass. To assess spatial variability in variance in biomass, we ran an ANOVA with the independent variable LFO. To test the seasonality of each variable at individual LFOs, we ran ANOVAs with month as the ordinal independent variable for each dependent variable: biomass; taxon richness; and proportion of top-trophic-level taxa.

In order to provide long-term context for seasonal trends in resilience indicators, we also analyzed long-term trends for each of the four resilience indicators at the spatial scale of Baja California Sur, where data from all ten LFOs were aggregated. For these analyses, we ran ANOVAs with year as the continuous independent variable for each dependent variable. All statistical analyses were conducted using JMP Pro 12 (SAS Institute Inc. 2015).

#### Correlations among indicators

To test whether these potential indicators of high ecological resilience are correlated with one another, we conducted a Pearson’s correlation for each pair of indicators at the spatial scale of the LFO.

#### Taxon-specific trends

For each LFO, the top taxa by biomass and value were determined as those taxa whose biomass or economic value made up the ten highest proportions of total annual biomass or value in an average year. There was considerable overlap between the top biomass and top value taxa. We excluded from temporal analyses those taxa with Spanish common names for which genera were unknown. To determine intra-annual variation in biomass of these taxa at each LFO, we conducted ANOVAs with month as an independent ordinal variable for each taxon. These analyses are reported in S1 Appendix.

## Results

### Spatial and temporal trends in resilience indicators

**Fig 2:**
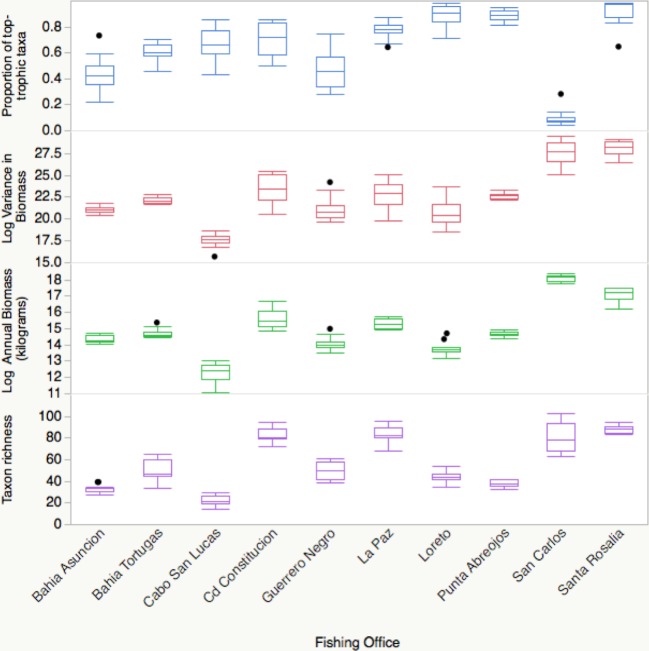
Ecological indicators vary by fishing office. Box and whisker plot showing significant spatial variation in all four ecological resilience indicators tested. Boxes represent 25^th^ to 75^th^ percentile for annual taxon proportion of top-trophic-level taxa, log variance in biomass, log total biomass, and taxon richness from 2001–2013, with points representing outliers. See Fig 1 for locations of the fishing offices.

All four ecological resilience indicators exhibited substantial spatial variation: the magnitude and variance of landed biomass [F(9, 1549) = 710.641, p<0.001; F(9, 120) = 104.613, p<0.001], taxon richness of reported catches [F(9, 1549) = 1302.497, p<0.001] and the proportion of top-trophic-level taxa [F(9, 1549) = 188.565, p<0.001] in reported landings all varied significantly among BCS’ LFOs for the time period studied (Fig 2). In addition, taxon composition of reported landings also varied markedly among the LFOs (Table 2).

**Table 2:** Taxa composition of total biomass and value for each fishing office.

Fishing office	Mean annual biomass (kilograms)	Mean annual taxon richness	Top 10 taxa by biomass (% of total biomass in an average year)	Top 10 taxa by value (% of total value in an average year)
Bahía Asunción	1,719,835	33	*Calappa spp* (27.5%); *Dosidicus gigas* (10.8%); *Cancer spp* (10.7%); *Panulirus interruptus* (9.3%); *Gelidium robustum* (5.8%); *Seriola lalandi* (3.9%); *Astraea undosa* (3.4%); *Prionace glauca* (2.7%); *Isostichopus fuscus* (1.7%); *Haliotis fulgens* (1.6%)	*Haliotis fulgens* (60.2%); *Panulirus interruptus* (16.6%); *Calappa spp* (6.6%); *Cancer spp* (3.7%); *Haliotis corrugate* (1.9%); *Isostichopus fuscus* (1.7%); *Gelidium robustum* (1.4%); *Astraea undosa* (1.3%); *Dosidicus gigas* (1.0%); *Seriola lalandi* (0.7%)
Bahía Tortugas	2,535,833	51	*Dosidicus gigas* (18.5%); *Gelidium robustum* (11.8%); *Octopus spp* (5.3%); *Katsuwonus pelamis* (4.0%); *Panulirus interruptus* (3.6%); *Strongylocentrotus franciscanus* (3.5%); *Crassostrea gigas* (3.2%); *Astraea undosa* (2.9%); *Panulirus spp* (2.7%); *Isostichopus fuscus* (2.6%)	*Strongylocentrotus franciscanus* (27.1%); *Panulirus interruptus* (14.1%); *Panulirus spp* (6.6%); *Octopus spp* (5.5%); *Haliotis fulgens* (4.2%); *Strongylocentrotus purpuratus* (4.1%); *Dosidicus gigas* (3.4%); *Crassostrea gigas* (3.2%); *Gelidium robustum* (3.1%); *Astraea undosa* (2.7%)
Cabo San Lucas	251,995	22	*Crassostrea iridescens* (14.3%); *Sphoeroides spp* (6.1%); *Dasyatis spp* (5.4%); *Lutjanus argentiventris* (5.3%); *Lutjanus peru* (5.2%); *Balistes polylepis* (4.7%); *Scomberomorus sierra* (4.1%); *Paranthias colonus* (4.0%); *Caulolatilus princeps* (3.7%); *Seriola lalandi* (3.3%)	*Lutjanus peru* (10.3%); *Sphoeroides spp* (7.0%); *Crassostrea iridescens* (5.0%); *Caranx sexfasciatus* (4.0%); *Dasyatis spp* (4.0%); *Hyporthodus niphobles* (3.9%); *Paranthias colonus* (3.7%); *Lutjanus guttatus* (3.7%); *Scomberomorus sierra* (3.3%); *Balistes polylepis* (3.0%)
Cd. Constitución	7,285,441	83	*Katsuwonus pelamis* (15.1%); *Dosidicus gigas* (14.1%); *Litopenaeus vannamei* (8.0%); *Chione undatella* (6.2%); *Argopecten circularis* (4.3%); *Crassostrea spp* (3.0%); *Hexaplex spp* (2.8%); *Paralabrax nebulifer* (2.4%); *Carcharinus limbatus* (2.3%); *Prionace glauca* (1.7%)	*Litopenaeus vannamei* (25.3%); *Katsuwonus pelamis* (8.3%); *Litopenaeus spp* (7.7%); *Panulirus inflatus* (6.3%); *Panulirus interruptus* (4.6%); *Haliotis fulgens* (4.1%); *Crassostrea spp* (2.3%); *Argopecten circularis* (1.7%); *Cancer spp* (1.7%); *Farfantepenaeus californiensis* (1.6%)
Guerrero Negro	1,412,877	50	*Dosidicus gigas* (35.8%); *Carcharhinus falciformis* (4.7%); *Paralabrax nebulifer* (4.6%); *Prionace glauca* (4.0%); *Panulirus interruptus* (4.0%); *Megapitaria squalida* (3.2%); *Caulolatilus princeps* (2.8%); *Paralabrax auroguttatus* (2.8%); *Crassostrea gigas* (1.7%); *Anadara spp* (1.7%)	*Panulirus interruptus* (40.4%); *Dosidicus gigas* (9.8%); *Panulirus spp* (5.4%); *Carcharhinus falciformis* (3.4%); *Crassostrea gigas* (2.3%); *Prionace glauca* (2.1%); *Tagelus californianus* (2.1%); *Spondylus spp* (2.0%); *Paralabrax nebulifer* (1.6%); *Diplectrum pacificum* (1.5%); *Paralabrax auroguttatus* (1.5%)
La Paz	4,545,882	83	*Katsuwonus pelamis* (20.1%); *Dosidicus gigas* (18.0%); *Litopenaeus spp* (11.1%); *Litopenaeus vannamei* (9.6%); *Prionace glauca* (2.4%); *Crassostrea spp* (2.1%); *Crassostrea iridescens* (2.0%); *Megapitaria squalida* (1.5%); *Argopecten circularis* (1.3%); *Anadara spp* (0.9%)	*Litopenaeus spp* (22.4%); *Litopenaeus vannamei* (21.3%); *Katsuwonus pelamis* (8.4%); *Panulirus inflatus* (5.5%); *Crassostrea spp* (4.9%); *Cherax quadicarinatus* (2.5%); *Farfantepenaeus californiensis* (2.1%); *Argopecten circularis* (1.6%); *Haliotis fulgens* (1.6%); *Prionace glauca* (1.2%)
Loreto	1,029,107	44	*Dosidicus gigas* (43.0%); *Alopias spp* (6.1%); *Carcharhinus limbatus* (5.0%); *Megapitaria squalida* (4.2%); *Alopias vulpinas* (3.1%); *Seriola lalandi* (1.4%); *Scomberomorus sierra* (1.4%); *Lutjanus peru* (1.3%); *Gnathodon spp* (1.1%); *Squatina californica* (1.1%)	*Dosidicus gigas* (8.1%); *Alopias spp* (6.4%); *Carcharhinus limbatus* (5.4%); *Lutjanus peru* (5.2%); *Lutjanus argentiventris* (4.7%); *Caranx sexfasciatus* (4.6%); *Alopias vulpinas* (3.0%); *Hyporthodus niphobles* (2.9%); *Scarus spp* (2.3%); *Megapitaria squalida* (1.9%)
Punta Abreojos	2,407,040	38	*Paralabrax nebulifer* (16.2%); *Panulirus interruptus* (16.0%); *Dosidicus gigas* (6.7%); *Seriola lalandi* (6.1%); *Crassostrea gigas* (6.0%); *Caranx caballus* (4.7%); *Astraea undosa* (4.6%); *Katsuwonus pelamis* (4.1%); *Synodus spp* (3.8%); *Xiphias gladius* (2.8%)	*Panulirus interruptus* (49.6%); *Haliotis corrugata* (10.6%); *Haliotis fulgens* (7.8%); *Crassostrea gigas* (5.0%); *Xiphias gladius* (4.3%); *Astraea undosa* (2.7%); *Panulirus inflatus* (2.3%); *Paralabrax nebulifer* (2.3%); *Seriola lalandi* (2.3%); *Crassostrea spp* (1.9%)
San Carlos	74,279,928	80	*Katsuwonus pelamis* (27.3%); *Caranx caballus* (17.2%); *Euthynnus pelamis* (10.3%); *Cancer spp* (7.5%); *Dosidicus gigas* (5.6%); *Chione undatella* (4.2%); *Argopecten circularis* (2.4%); *Prionace glauca* (2.2%); *Xiphias gladius* (1.6%); *Sicyonia dorsalis* (1.2%)	*Katsuwonus pelamis* (21.8%); *Caranx caballus* (18.6%); *Cancer spp* (9.6%); *Xiphias gladius* (9.4%); *Euthynnus lineatus* (8.5%); *Panulirus inflatus* (3.4%); *Argopecten circularis* (1.3%); *Panulirus interruptus* (1.3%); *Prionace glauca* (1.2%); *Dosidicus gigas* (1.2%)
Santa Rosalía	28,746,044	88	*Dosidicus gigas* (31.1%); *Trachurus symmetricus* (9.0%); *Crassostrea gigas* (5.0%); *Synodus spp* (3.8%); *Crassostrea spp* (3.3%); *Paralabrax nebulifer* (3.0%); *Megapitaria squalida* (2.7%); *Tivela stultorum* (2.2%); *Xiphias gladius* (2.2%); *Alopias spp* (2.1%)	*Panulirus interruptus* (11.3%); *Crassostrea gigas* (9.0%); *Dosidicus gigas* (7.0%); *Crassostrea spp* (5.9%); *Octopus spp* (2.9%); *Alopias spp* (2.7%); *Synodus spp* (2.5%); *Atrina spp* (2.4%); *Paralabrax nebulifer* (2.1%); *Megapitaria squalida* (2.1%)

Taxa composition of total biomass and value for 10 fishing offices in Baja California Sur, from 2001–2013.

**Fig 3:**
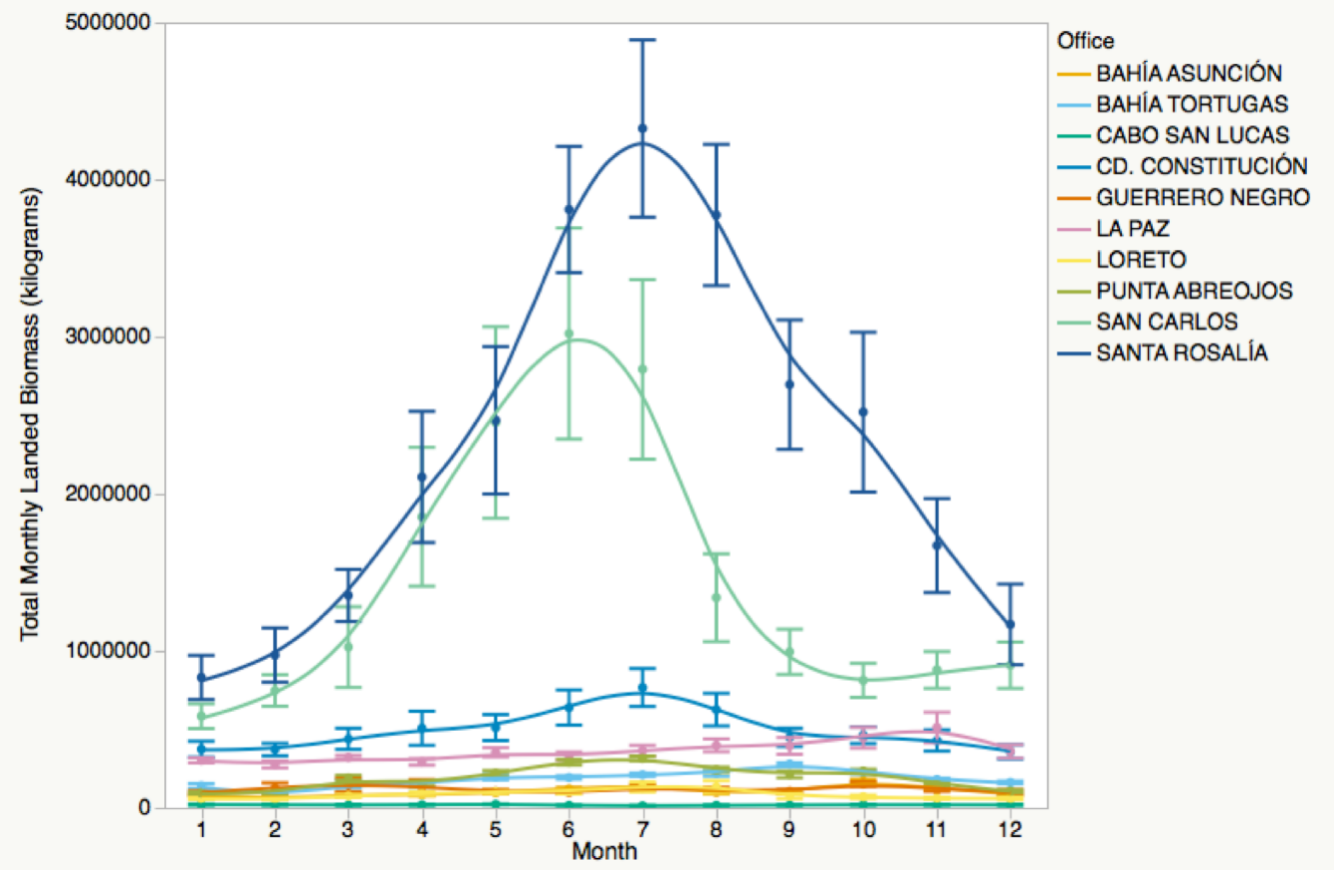
Landed biomass by month for all ten fishing offices. Intra-annual variation in total fisheries biomass based on data reported to CONAPESCA by small-scale fishers from 2001–2013 for all ten offices in Baja California Sur, Mexico. Error bars represent one standard error from the mean.

**Fig 4:**
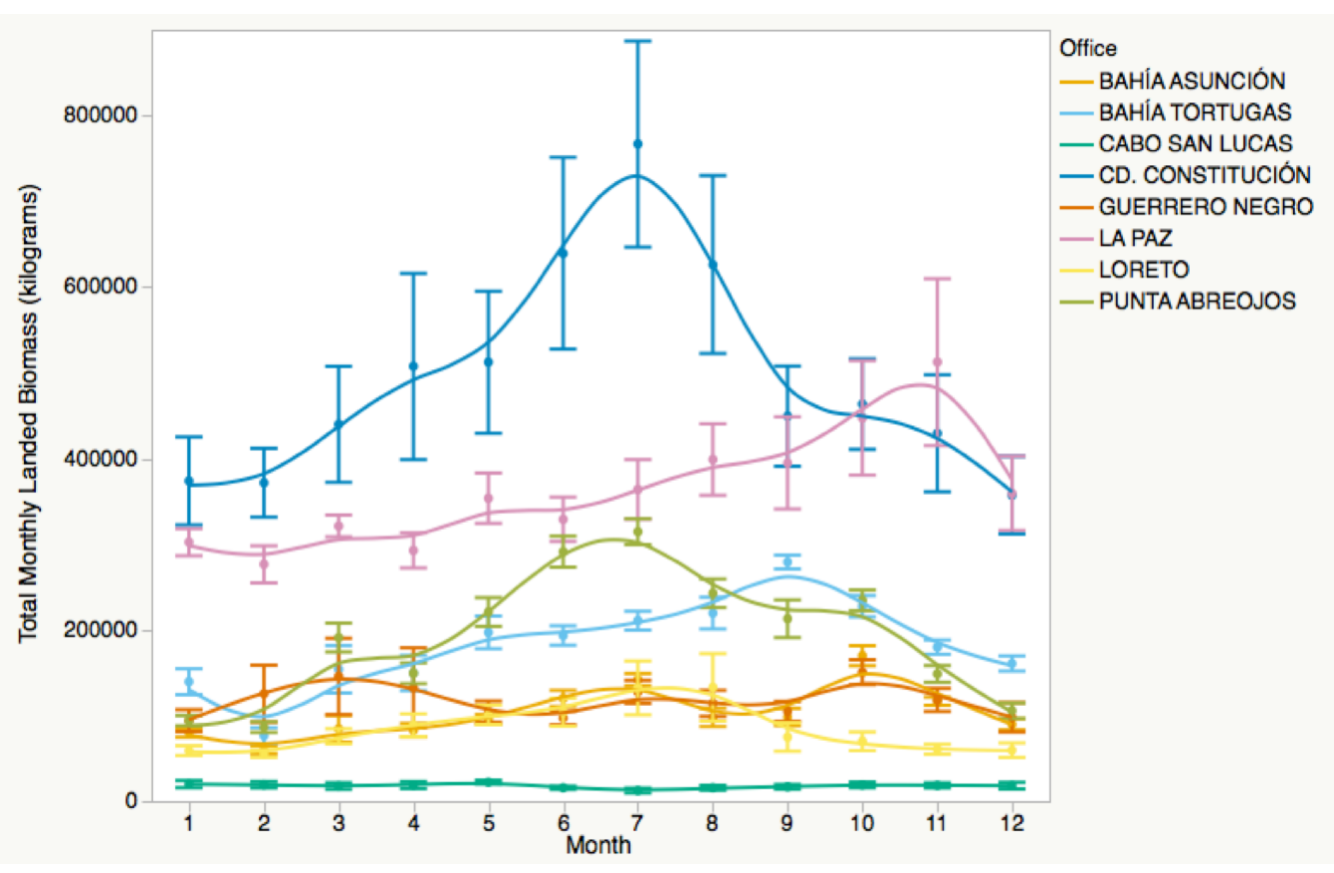
Landed biomass by month for eight of ten fishing offices. Intra-annual variation in total fisheries biomass based on data reported to CONAPESCA by small-scale fishers from 2001–2013 for eight offices in Baja California Sur, Mexico. Error bars represent one standard error from the mean. Top biomass offices Santa Rosalía and San Carlos are excluded to better show trends in other offices.

We found significant intra-annual variation in total landed biomass, and each LFO experienced this variation differently [Fig 3; F(119, 1439) = 69.531, p<0.001]. Exclusion of top biomass LFOs, San Carlos and Santa Rosalía, did not change these results, but made it easier to visually assess among-office differences in the remaining eight offices (see Fig 4).

We found significant intra-annual variation in both taxon richness and proportion of total landings made up of top-trophic-level taxa, with LFOs experiencing differential seasonal fluctuations in both indicators [F(119, 1439) = 102.711, p<0.001; F(119,1439) = 22.536, p<0.001].

Long-term analyses at the scale of BCS revealed an increase in both total landed biomass and proportion of top-trophic-level taxa from 2001–2013 [F(1,1557) = 5.593, p = 0.018; F(1,1557) = 9.308, P = 0.002], but no directional long-term trend in the other two indicators: variance in biomass [F(1,128) = 0.329, p = 0.567], and taxon richness [F(1,1557) = 0.703, p = 0.402]. Complete results of these analyses can be found in S2 Appendix.

### Correlations among indicators

We found significant positive correlation between several of the resilience indicators tested. Taxon richness increased with increasing total biomass (r = 0.831, r^2^ = 0.691, p<0.001), and with increasing variance in biomass (r = 0.752, r^2^ = 0.566, p<0.001). We also found that taxon richness increased with increasing proportion of top-trophic-level taxa (r = 0.166, r^2^ = 0.028, p<0.001), as predicted. Variance in biomass also increased with increasing total biomass of landings (r = 0.952, r^2^ = 0.906, p<0.001). However, we found no relationship between proportion of top-trophic-level taxa and either total biomass of landings (r = 0.004, r^2^< 0.001, p = 0.874) or variance in biomass (r = 0.128, r^2^ = 0.016, p = 0.146).

### Taxon-specific and resilience indicator trends within individual LFOs

LFOs varied in taxon richness, total biomass and composition of landings (Table 3). Here we discuss some of the trends. For a full description, please see S1 Appendix. We focus on four of the ten offices, because of their importance to the marine economy of BCS, as well as the biogeographic variation they represent.

**Table 3:** Ecological indicators vary by fishing office.

Fishing Office	Mean annual biomass (kilograms)	Mean variance in biomass among months	Mean monthly taxon richness	Mean monthly proportion of top-trophic-level taxa in total landings
Bahía Asunción	1,699,364	1.76 x 10^9	20.4	0.56
Bahía Tortugas	2,493,535	5.22 x 10^9	28.7	0.68
Cabo San Lucas	251,995	1.33 x 10^8	9.0	0.85
Cd. Constitución	7,285,441	9.23 x 10^10	50.0	0.81
Guerrero Negro	1,412,877	7.16 x 10^9	24.8	0.45
La Paz	4,545,882	2.87 x 10^10	51.0	0.80
Loreto	1,029,107	4.41 x 10^9	27.5	0.89
Punta Abreojos	2,407,040	7.61 x 10^9	27.5	0.93
San Carlos	74,279,948	2.25 x 10^12	46.9	0.56
Santa Rosalía	28,746,044	3.01 x 10^12	57.8	0.97

Summary of the ecological resilience indicators, by fishing office. Mean variance in biomass is calculated as the mean annual variance in monthly biomass from 2001–2013.

#### San Carlos

In San Carlos, the most productive LFO in terms of both total landed biomass and total value of landings, we found that biomass of landings and proportion of top-trophic-level taxa varied intra-annually [F(11, 144) = 5.364, p<0.001; F(11, 144) = 2.888, p = 0.002 respectively], while taxon richness did not [F(11, 144) = 1.102, p = 0.364]. Top taxa exhibited low seasonality, indicating availability to fishers throughout the year. The two top taxa in San Carlos by biomass and value, *Katsuwonus pelamis* (Skipjack tuna) and *Caranx caballus* (Green jack) showed no significant seasonality, while other top taxa exhibited variable seasonal trends; *Panulirus inflatus* (Blue spiny lobster) and *Panulirus interruptus* (California spiny lobster), two of this LFO’s highest-value taxa, both peaked October through December, while another top taxa, *Argopecten circularis* (Catarina scallop) peaked May through July. Thus, while we found evidence of seasonality in San Carlos, the stability of both taxon richness and landings of top taxa indicates consistent ecological resilience throughout the year.

#### Santa Rosalía

In Santa Rosalía, the second most productive LFO in terms of reported total biomass, total landed biomass varied significantly among months [F(11, 144) = 7.007, p<0.001] while taxon richness remained constant [F(11, 144) = 0.739, p = 0.700]. Proportion of top-trophic-level taxa also varied throughout the year, with significantly lower proportions of top-trophic-level taxa in January and February [F(11, 144) = 5.205, p<0.001].

Top taxa landed in Santa Rosalía exhibited mixed seasonality, with *Dosidicus gigas* (Giant squid) and *Octopus spp* (octopus) peaking May to August and *Paralabrax nebulifer* (Barred sand bass) peaking in August through March. *Atrina spp* (scallop) and *Panulirus interruptus* (California spiny lobster) peaked in February and September and October to November, respectively. Meanwhile, other important taxa including *Trachurus symmetricus* (Pacific jack mackerel), *Alopias spp* (thresher shark), *Megapitaria squalida* (chocolate clam) and *Crassostrea gigas* (Pacific oyster), appeared in the landings data consistently throughout the year and showed no seasonal trends, providing fishers with a reliable resource when other target taxa were not in season.

#### La Paz

In La Paz, the most populated area of BCS in terms of total fishermen, other than San Carlos on the Pacific coast [20], we observed no intra-annual variation in total landed biomass [F(11,144) = 1.624, p = 0.098]. We also found no intra-annual variation in either taxon richness [F(11,144) = 0.703, p = 0.734] or proportion of top-trophic-level taxa [F(11,144) = 0.995, p = 0.454]. Only three of La Paz’s top taxa exhibited significant seasonality; the majority of target taxa were harvested throughout the year. Seasonal taxa included *Litopenaeus spp* (shrimp), *Litopenaeus vannamei* (Whiteleg shrimp), and *Crassostrea iridesens* (Rock oyster), which all peaked September to December.

#### Punta Abreojos

In Punta Abreojos, one of the areas of BCS with a reputation for strong fisheries governance [20,24], we observed intra-annual variation in total biomass [F(11, 144) = 33.521, p<0.001] and proportion of top-trophic-level taxa [F(11, 144) = 11.231, p<0.001], but no intra-annual variation in taxon richness [F(11, 144) = 1.539, p = 0.124]. Unlike San Carlos, in which few top taxa exhibited seasonality, Punta Abreojo’s top taxa were highly seasonal: *Haliotis spp* (abalone), *Dosidicus gigas* (Giant squid) and *Paralabrax nebulifer* (Barred sand bass) all peaked March to June; *Caranx caballus* (Green jack) and *Seriola lalandi* (Yellowtail) peaked July to September; *Astraea undosa* (sea snail) and *Panulirus interruptus* (California spiny lobster) peaked September to November; and *Panulirus inflatus* (Blue spiny lobster) peaked November to January. Thus, while richness of landings remained constant year-round, composition varied seasonally.

## Discussion

For the 13 years of landings data used in this study, we found that the magnitude and variance of landed biomass, taxon richness of reported catches, and the proportion of top-trophic-level taxa in reported catches all varied spatially within BCS, supporting our hypothesis that the four variables exhibit spatial variation. Moreover, in support of the second hypothesis, we also found significant seasonal variation in magnitude of landed biomass, taxon richness of reported catches, and the proportion of landed biomass made up of top-trophic-level taxa. These trends were not evident from state-level analyses, contrary to our expectations (see S2 Appendix for details).

Local fishing office (LFO)-specific trends in the four resilience indicators we assessed varied considerably. Baja California Sur encompasses several diverse social-ecological system regions, defined by the oceanographically and ecologically-distinct Pacific and Gulf of California coasts, as well as diverse fisher and institutional characteristics [20,25]. Spatial variability in resilience indicators among LFOs could also be attributed to such factors as the number of fishers reporting, economic reliance on fishing, and fleet diversity. LFOs in regions with large human populations reported high biomass of catch (*e*.*g*., Cd. Constitución, La Paz, San Carlos, Santa Rosalía), except where small-scale fishing is less important than other economic activities, such as tourism (*e*.*g*., Cabo San Lucas). High taxon diversity of catch may be related to high fishing fleet and gear diversity, where diverse gear types allow fishers to target a greater variety of taxa. This spatial variability highlights the importance of LFO-level dynamics in understanding how fisheries trends impact both the ecological and human dimensions of these coupled systems. Further, our finding of seasonal variability in ecological indicators emphasizes the necessity of conducting such studies at time scales relevant to fishers’ experiences.

The potential indicators of ecological resilience we examined did not always correspond with one another in ways consistent with theory, nor in the ways we hypothesized. For example, high proportions of top-trophic-level taxa in landed biomass, theoretically associated with high ecological resilience [7,12], were not necessarily found alongside low variance in biomass, another indicator of high resilience, as predicted [26,27]. The context dependency of resilience indicators, where landings can suggest high resilience in one indicator and low resilience in another, further emphasizes the importance of small-scale dynamics and further investigations to understand the fisheries SESs of this region.

Changes in ecological variables, including the resilience indicators we focus on here, impact the ability of ecosystems, or SESs, to maintain functioning in the face of change. We describe key variables for a particular window of time, rather than examining social-ecological interactions or the outcomes that flow from these interactions. While our study provides a static description and serves as a foundational step towards understanding spatial and temporal patterns, more dynamic approaches (*e*.*g*., agent-based modeling, after [28]) are needed to understand SES interactions and outcomes [29] and to forecast fisheries and fishermen’s responses to climate variability and change [30,31].

Spatial and seasonal variation in taxon composition, biomass and richness may provide unique opportunities for fishers to adapt to the increasing variability predicted with climate change [15,32]. Fishers may adapt to variability by migrating seasonally to areas with greater fishing opportunity, by diversifying their catch at certain times of the year, or by adopting additional livelihood strategies [33]. Fishers regularly employ these strategies in BCS [15,34]. Diversification of fishing catch has been shown to mitigate fishers’ economic risk by reducing income variation [33,35,36]. Thus, environmental variability that would ordinarily be expected to stress coastal communities, may serve to enhance resilience by diversifying fishers’ opportunities and facilitating adaptation [37]. Understanding seasonal changes and fishers’ responses to them is crucial to the design of temporally-appropriate fisheries management [30,32].

Fisheries-dependent data such as those used in this study have a number of limitations. First, they are limited to taxa targeted by fishers. Taxa composition of fisheries is not directly equivalent to assemblages of marine taxa *in situ*, and biomass of landings is affected by fishing effort and market demand [7]. We therefore do not know whether the temporal trends in biomass of landings observed in this study resulted from changes in the populations of fished taxa or from changes in fishing effort, such as from periodic fishing bans (known in Spanish as *vedas*). Unfortunately, landings data for small-scale fisheries in BCS are often inadequately reported, and due to the nature of reporting, reliable data on fishing effort do not exist [38]. In the future, we could address this issue by using human population and fishing vessel density to predict the spatial distribution of fishing effort [39]. Social science efforts, including fisher participatory approaches, may also be useful for estimating fishing effort in this region.

Despite its limitations, the data used in this study provide information on the dynamics of numerous taxa for which monitoring either has not been done or is not feasible. Fisheries-dependent data are influenced by both ecological and socio-economic dynamics, and thus provide valuable information for studying the dynamics of human resource use. This study provides insight into how to operationalize assessments of resilience in social-ecological systems, and in fisheries SESs specifically. Ideally, ecological resilience is assessed based on both fishery independent and dependent data, but in many cases, historical landings data are often the only available information regarding trends in fished taxa [8]. Exploring how these data can be used to estimate the resilience of associated marine ecosystems can help scientists evaluate the health of these systems [3], and contribute to improved fisheries management.

Our results indicate that office-level variability is more significant than variability at the spatial scale of BCS. BCS fisheries are currently managed at the level of the state, ignoring significant spatial variability in fishing offices with differing ecological conditions and fished assemblages. Although Mexico’s national fisheries law permits greater decentralization of fisheries governance that would allow for place-specific management, this law is not yet fully functional in BCS [20]. We find that ecological indicators relevant to resilience vary spatially among fishing offices, and temporally within years, but are relatively stable over the 13 years of the dataset. Our findings highlight the value of managing fisheries at a finer spatial scale, recognizing that matching the scales of human activity and environmental dynamics is vital for sustaining marine fisheries [20,40].

## Supporting information

S1 Appendix Additional analyses for each fishing office. (PDF)

S2 Appendix Temporal trends in ecological resilience indicators at the spatial scale of Baja California Sur. (PDF)

S1 Dataset CONAPESCA landings data for Baja California Sur from 2001–2013. (CSV)
