# Supplementary material for: Seasonal variability shapes resilience of small-scale fisheries in Baja California Sur, Mexico
Source: PLoS One. 2017 Aug 4;12(8):e0182200. doi: 10.1371/journal.pone.0182200 (PMC5544237; doi:10.1371/journal.pone.0182200)
Supplement: S1 Appendix — (PDF) [file pone.0182200.s001.pdf]

S1 Appendix

Additional Analyses for each Fishing Office

Here we provide additional analyses for each office, including results of ANOVA tests of intra-annual variation for three of the four resilience indicators. Intra-annual analyses on variance in biomass were not possible given that variance was calculated across months. Offices are listed alphabetically.

Bahía Asunción

In Bahía Asunción, we observed intra-annual variation in biomass [$F(11, 144)=10.244$, $p<0.001$], taxon richness [$F(11, 144)=2.110$, $p=0.023$], and proportion of top-trophic-level taxa [$F(11, 144)=10.771$, $p<0.001$]. Table 1 gives monthly trends in the landings of top taxa.

Table 1.

Taxon name	Variation among months? (ANOVA)	Peak months
Haliotis corrugata	No, p=0.120	None
Haliotis fulgens	Yes, p<0.001	Apr-Jun
Gelidium robustum	Yes, p<0.001	Mar-Nov
Dosidicus gigas	No, p=0.408	None
Cancer spp	No, p=0.873	None
Calappa spp	No	None
Astraea undosa	No, p=0.335	None
Seriola lalandi	Yes, p<0.001	Aug-Nov
Panulirus interruptus	Yes, p<0.001	Oct
Isostichopus fuscus	Yes, p=0.001	Jun-Jul
Prionace glauca	No, p=0.306	None

Long-term trends from 2001-2013 in landed biomass of top taxa in Bahía Asunción.

Bahía Tortugas

We found intra-annual variation in biomass [F(11, 144)=18.046, p<0.001], taxon richness [F(11, 144)=4.973, p<0.001], and in the proportion of top-trophic-level taxa [F(11, 144)=24.448, p<0.001]. Table 2 gives monthly trends in the landings of top taxa.

Table 2.

Taxon name	Variation among months? (ANOVA)	Peak months
Haliotis fulgens	Yes, p<0.001	April-May
Gelidium robustum	Yes, p<0.001	May-Aug
Katsuwonus pelamis	No	None
Dosidicus gigas	No, p=0.643	None
Astraea undosa	Yes, p=0.001	April-Aug
Strongylocentrotus purpuratus	No	None
Strongylocentrotus franciscanus	No, p=0.264	None
Panulirus spp	No	None
Panulirus interruptus	Yes, p<0.001	Sept-Dec
Crassostrea gigas	No, p=0.566	None
Isostichopus fuscus	Yes, p<0.001	June-July
Octopus spp	No, p=0.184	None

Long-term trends from 2001-2013 in landed biomass of top taxa at the spatial scale of Bahía Tortugas.

Cabo San Lucas

In Cabo San Lucas, with the lowest reported biomass and the lowest taxon diversity over the data we analyzed, we documented no intra-annual variation in landed biomass [F(11, 144)=0.577, p=0.845] or in taxon richness [F(11, 144)=0.522, p=0.886]. We found intra-annual variation in the proportion of top-trophic-level taxa [F(11, 144)=0.221, p=0.016]. Table 3 gives monthly trends in the landings of top taxa.

Table 3.

Taxon name	Variation among months? (ANOVA)	Peak months
Sphoeroides spp	No, p=0.947	None
Paranthias colonus	No, p=0.413	None
Balistes polylepis	No, p=0.664	None
Hyporthodus niphobles	No, p=0.746	None
Lutjanus peru	No, p=0.319	None
Seriola lalandi	No, p=0.871	None
Lutjanus guttatus	No, p=0.593	None
Dasyatis spp	No, p=0.423	None
Caranx sexfasciatus	No, p=0.808	None
Crassostrea iridescens	No, p=0.213	None
Lutjanus argentiventris	No, p=0.067	None
Caulolatilus princeps	No, p=0.284	None
Scomberomorus sierra	No, p=0.814	None

Long-term trends from 2001-2013 in landed biomass of top taxa in Cabo San Lucas.

Cd. Constitución

We observed intra-annual variation in biomass [F(11, 144)=2.014, p=0.031], but found no intra-annual variation in either taxon richness [F(11, 144)=0.314, p=0.982] or in the proportion of top-trophic-level taxa [F(11, 144)=1.645, p=0.092]. Table 4 gives monthly trends in the landings of top taxa.

Table 4.

Taxon name	Variation among months? (ANOVA)	Peak months
Haliotis fulgens	No, p=0.351	None
Argopecten circularis	No, p=0.533	None
Chione undatella	No, p=0.417	None
Katsuwonus pelamis	No, p=0.145	None
Dosidicus gigas	No, p=0.588	None
Litopenaeus spp	No, p=0.504	None
Litopenaeus vannamei	No, p=0.303	None
Farfantepenaeus californiensis	Yes, p=0.002	Jan, Sept
Cancer spp	No	None
Hexaplex spp	No, p=0.893	None
Panulirus inflatus	No, p=0.072	None
Panulirus interruptus	Yes, p<0.001	Oct-Nov
Crassostrea spp	No, p=0.143	None
Prionace glauca	No, p=0.593	None
Carcharhinus limbatus	No, p=0.762	None
Paralabrax nebulifer	Yes, p<0.001	Jul-Aug

Long-term trends from 2001-2013 in landed biomass of top taxa in Cd. Constitución.

Guerrero Negro

We found no intra-annual variation in biomass [F(11, 144)=1.142, p=0.333] or in proportion of top-trophic-level taxa [F(11, 144)=1.627, p=0.097], but did observe intra-annual variation in taxon richness [F(11, 144)=2.909, p=0.002]. Table 5 gives monthly trends in the landings of top taxa.

Table 5.

Taxon name	Variation among months? (ANOVA)	Peak months
Megapitaria squalida	Yes, p<0.001	Feb, Oct
Spondylus spp	No	None
Tagelus californianus	No, p=0.296	None
Anadara spp	No, p=0.884	None
Diplectrum pacificum	Yes, p=0.041	Mar-Apr
Dosidicus gigas	No, p=0.513	None
Paralabrax auroguttatus	Yes, p=0.021	Jan-Mar
Panulirus spp	No	None
Panulirus interruptus	Yes, p<0.001	Sept-Nov
Crassostrea gigas	No, p=0.815	None
Caulolatilus princeps	Yes, p=0.001	July (highest), Oct
Prionace glauca	No, p=0.400	None
Carcharhinus falciformis	No	None
Paralabrax nebulifer	Yes, p=0.005	July-Aug

Long-term trends from 2001-2013 in landed biomass of top taxa in Guerrero Negro.

La Paz

We observed no intra-annual variation in biomass [F(11, 144)=1.624, p=0.098], taxon richness [F(11, 144)=0.703, p=0.734], or proportion of top-trophic-level taxa [F(11, 144)=0.995, p=0.454]. Table 6 gives monthly trends in the landings of top taxa.

Table 6.

Taxon name	Variation among months? (ANOVA)	Peak months
Haliotis fulgens	No, p=0.618	None
Argopecten circularis	No, p=0.805	None
Megapitaria squalida	No, p=0.768	None
Anadara spp	No, p=0.862	None
Katsuwonus pelamis	No	None
Dosidicus gigas	No, p=0.967	None
Litopenaeus spp	Yes, p=0.002	Sept-Nov
Litopenaeus vannamei	Yes, p=0.008	Sept-Dec
Farfantepenaeus californiensis	No, p=0.578	None
Panulirus inflatus	No, p=0.701	None
Cherax quadicarinatus	No	None
Crassostrea spp	No, p=0.573	None
Crassostrea iridescens	Yes, p=0.004	Oct-Nov
Prionace glauca	No, p=0.897	None

Long-term trends from 2001-2013 in landed biomass of top taxa in La Paz.

Loreto

In the fishing office of Loreto, we observed intra-annual variation in biomass [$F(11, 144)=2.916$, $p=0.002$], but found that neither taxon richness nor proportion of top-trophic-level taxa varied among months [$F(11, 144)=0.961$, $p=0.485$; $F(11, 144)=0.536$, $p=0.876$ respectively]. Table 7 gives monthly trends in the landings of top taxa.

Table 7.

Taxon name	Variation among months? (ANOVA)	Peak months
Megapitaria squalida	No, p=0.687	None
Squatina californica	Yes, p=0.012	Nov-Jan
Dosidicus gigas	No, p=0.385	None
Hyporthodus niphobles	No, p=0.335	None
Lutjanus peru	Yes, p<0.001	May-Oct
Seriola lalandi	Yes, p=0.025	Mar, June
Caranx sexfasciatus	No, p=0.235	None
Gnathodon spp	No, p=0.861	None
Lutjanus argentiventris	Yes, p<0.001	May
Scarus spp	No, p=0.308	None
Scomberomorus sierra	No, p=0.507	None
Alopias spp	No, p=0.226	None
Carcharhinus limbatus	No, p=0.110	None
Alopias vulpinas	No, p>0.05	None

Long-term trends from 2001-2013 in landed biomass of top taxa in Loreto.

Punta Abreojos

In the fishing office of Punta Abreojos, we observed intra-annual variation in total biomass [F(11, 144)=33.521, p<0.001], and in proportion of top-trophic-level taxa [F(11, 144)=11.231, p<0.001], but we found no intra-annual variation in taxon richness [F(11, 144)=1.539, p=0.124]. Table 8 gives monthly trends in the landings of top taxa.

Table 8.

Taxon name	Variation among months? (ANOVA)	Peak months
Haliotis corrugata	Yes, p<0.001	Mar-Jun
Haliotis fulgens	Yes, p<0.001	Mar-Jun
Katsuwonus pelamis	No	None
Caranx caballus	Yes, p<0.001	Jul-Aug
Dosidicus gigas	Yes, p=0.020	Mar, May
Astraea undosa	Yes, p=0.015	March, Aug-Nov
Synodus spp	No	None
Seriola lalandi	Yes, p<0.001	Jul-Sept
Panulirus inflatus	Yes, p<0.001	Nov-Jan
Panulirus interruptus	Yes, p<0.001	Oct-Nov
Crassostrea spp	No, p=0.479	None
Crassostrea gigas	No, p=0.712	None
Xiphias gladius	No, p=0.461	None
Paralabrax nebulifer	Yes, p<0.001	March-June

Long-term trends from 2001-2013 in landed biomass of top taxa in Punta Abreojos.

San Carlos

We observed intra-annual variation in biomass [$F(11, 144)=5.364$, $p<0.001$], and in proportion of top-trophic-level taxa [$F(11, 144)=2.888$, $p=0.002$], but we found no intra-annual variation in taxon richness [$F(11, 144)=1.102$, $p=0.364$]. Table 9 gives monthly trends in the landings of top taxa.

Table 9.

Taxon name	Variation among months? (ANOVA)	Peak months
Argopecten circularis	Yes, p<0.001	May-July
Chione undatella	No, p=0.329	None
Katsuwonus pelamis	No, p=0.581	None
Euthynnus lineatus	No, p=0.486	None
Caranx caballus	No, p=0.903	None
Dosidicus gigas	No, p=0.534	None
Sicyonia dorsalis	No, p=0.803	None
Cancer spp	No, p=0.620	None
Panulirus inflatus	Yes, p<0.001	Nov-Dec
Panulirus interruptus	Yes, p=0.005	Oct-Dec
Xiphias gladius	Yes, p<0.001	Oct-April
Prionace glauca	No, p=0.282	None

Long-term trends from 2001-2013 in landed biomass of top taxa in San Carlos.

Santa Rosalía

In the fishing office of Santa Rosalía, we found intra-annual variation in biomass [$F(11, 144)=7.007$, $p<0.001$], and in the proportion of top-trophic-level taxa [$F(11, 144)=5.205$, $p<0.001$], but we found no intra-annual variation in taxon richness [$F(11, 144)=0.739$, $p=0.700$]. Table 10 gives monthly trends in the landings of top taxa.

Table 10.

Taxon name	Variation among months? (ANOVA)	Peak months
Megapitaria squalida	No, p=0.380	None
Tivela stultorum	No, p=0.695	None
Dosidicus gigas	Yes, p<0.001	Peak June-Aug
Atrina spp	Yes, p<0.001	Peak Feb, Sept
Trachurus symmetricus	No, p=0.547	None
Synodus spp	No	None
Panulirus interruptus	Yes, p<0.001	Peak Oct-Nov
Crassostrea spp	No, p=0.678	None
Crassostrea gigas	No, p=0.760	None
Xiphias gladius	No	None
Octopus spp	Yes, p<0.001	Peak May-Aug
Alopias spp	No, p=0.969	None
Paralabrax nebulifer	Yes, p<0.001	Peak Aug-Mar

Long-term trends from 2001-2013 in landed biomass of top taxa in Santa Rosalía.
