# Supplementary material for: Seasonal variability shapes resilience of small-scale fisheries in Baja California Sur, Mexico
Source: PLoS One. 2017 Aug 4;12(8):e0182200. doi: 10.1371/journal.pone.0182200 (PMC5544237; doi:10.1371/journal.pone.0182200)
Supplement: S2 Appendix — (PDF) [file pone.0182200.s002.pdf]

S2 Appendix

Temporal Trends in Resilience Indicators at the Spatial Scale of Baja California Sur

Taxa composition of reported fisheries landings varied both inter- and intra-annually at the scale of the Mexican state of BCS. The top five taxa with the highest reported biomass on average from 2001-2013 at the scale of BCS included *Dosidicus gigas* (Giant squid), *Argopecten ventricosus* (Catarina scallop), *Paralabrax nebulifer* (Barred sand bass), *Seriola lalandi* (Yellowtail), and *Panulirus interruptus* (California spiny lobster). BCS as a whole experienced a steady increase in total landed biomass of landings up to a peak in 2008. In 2009, there was a marked drop in total biomass of landings, an uptick in 2010, and a steady decrease in total landings thereafter until 2013. Total landed biomass increased slightly from 2001-2013 [$F(1, 1557)=5.593$, $p=0.018$], while variance in biomass exhibited no change over the same time period [$F(1, 128)=0.329$, $p=0.567$].

We found that for BCS as a whole, taxon richness peaked in the year 2010, but showed no directional trend from 2001-2013 [$F(1, 1557)=0.703$, $p=0.402$]. Taxon richness also did not vary among months at the spatial scale of BCS [$F(11, 1547)=0.2914$, $p=0.988$]. We found significant inter- and intra-annual variation in proportions of top-trophic-level taxa at the BCS spatial scale [$F(1, 1557)=9.3084$, $p=0.002$; $F(11, 1547)=5.495$, $p<0.001$ respectively], with proportion of top-trophic-level taxa increasing from 2001-2013 [$F(1,1557)=9.308$, $P=0.002$].
